# A novel reporter for helicase activity in translation uncovers DDX3X interactions

**DOI:** 10.1261/rna.079837.123

**Published:** 2024-08

**Authors:** Kevin C. Wilkins, Till Schroeder, Sohyun Gu, Jezrael L. Revalde, Stephen N. Floor

**Affiliations:** 1 Department of Cell and Tissue Biology, University of California, San Francisco, San Francisco, California 94143, USA; 2 Graduate Division, University of California, San Francisco, San Francisco, California 94143, USA; 3 Faculty of Chemistry and Pharmacy, Julius-Maximilians-University of Würzburg, Würzburg 97070, Germany; 4 Department of Pharmaceutical Chemistry, University of California, San Francisco, California 94143, USA; 5 Helen Diller Family Comprehensive Cancer Center, University of California, San Francisco, San Francisco, California 94143, USA

**Keywords:** RNA helicases, RNA structure, reporter genes, translational control

## Abstract

DDX3X regulates the translation of a subset of human transcripts containing complex 5′ untranslated regions (5′ UTRs). In this study, we developed the helicase activity reporter for translation (HART), which uses DDX3X-sensitive 5′ UTRs to measure DDX3X-mediated translational activity in cells. To directly measure RNA structure in DDX3X-dependent mRNAs, we used SHAPE-MaP to determine the secondary structures present in DDX3X-sensitive 5′ UTRs and then used HART to investigate how sequence alterations influence DDX3X sensitivity. Additionally, we identified residues 38–44 as potential mediators of DDX3X's interaction with the translational machinery. HART revealed that both DDX3X's association with the translational machinery and its helicase activity are required for its function in promoting the translation of DDX3X-sensitive 5′ UTRs. These findings suggest DDX3X plays a crucial role in regulating translation through its interaction with the translational machinery during ribosome scanning and establish the HART reporter as a robust, lentivirally encoded, colorimetric measurement of DDX3X-dependent translation in cells.

## INTRODUCTION

DDX3X is a ubiquitously expressed RNA helicase implicated in almost all stages of RNA metabolism (Soto-Rifo and Ohlmann 2013; Sharma and Jankowsky 2014). By influencing gene expression, RNA localization, and RNA stability, DDX3X impacts cellular functions and responses, including homeostasis (Tsai et al. 2017), stress granule regulation (Shih et al. 2012; Valentin-Vega et al. 2016), cell stress (Oh et al. 2016; Samir et al. 2019), signaling (Sharma and Jankowsky 2014), the immune response (Soulat et al. 2008; Szappanos et al. 2018), and embryonic development (Chen et al. 2016a; Lennox et al. 2020). Dysfunctions in DDX3X are linked with several human diseases, including cancer, viral infection, inflammation, and intellectual disabilities (Sharma and Jankowsky 2014; Gadek et al. 2023).

DDX3X is a member of the DEAD-box RNA helicase family and hydrolyzes ATP to unwind RNA molecules and remodel RNA–protein complexes (Linder and Jankowsky 2011). DDX3X shows high conservation with its yeast homolog Ded1, with which it forms a clearly defined subfamily (Fairman-Williams et al. 2010). DDX3X/Ded1 helicases are characterized by a conserved helicase core domain that contains nine motifs involved in ATP binding, hydrolysis, and RNA interaction (Linder and Jankowsky 2011; Sharma and Jankowsky 2014). Although the helicase core is highly conserved across the DEAD-box family, the N and C termini are variable (Linder and Jankowsky 2011; Floor et al. 2016). These termini are involved in protein–protein interactions and subcellular localization, but their role is yet to be fully understood (Sharma and Jankowsky 2014).

DDX3X plays a role in translation by unwinding RNA secondary structures and remodeling RNA–protein complexes. DDX3X/Ded1 have been suggested to interact with components of the translation initiation machinery, including the eIF4F complex (eIF4E, eIF4G, and eIF4A), to promote ribosome binding to the mRNA's 5′ cap and enhance the recruitment of ribosomes to the mRNA (Shih et al. 2012; Sharma and Jankowsky 2014; Guenther et al. 2018; Gupta et al. 2018; Gulay et al. 2020). During ribosome scanning, DDX3X unwinds secondary structures within the 5′ untranslated regions (UTRs) to facilitate the movement of ribosomes along the mRNA to allow for identification of the start codon (Soto-Rifo and Ohlmann 2013; Calviello et al. 2021). As opposed to eIF4A, a generalist necessary for global translation, DDX3X has been observed to act as specialist helicase, required for the proper translation of a subset of the human mRNAs (Linder and Jankowsky 2011; Calviello et al. 2021). This specialized role could derive from DDX3X/Ded1's higher RNA unwinding capability compared to eIF4A, suggesting that DDX3X might be required to resolve complex and highly structured 5′ UTRs (Gao et al. 2016).

Several aspects of DDX3X's activity during ribosome scanning and translation are still poorly understood. The complete repertoire of its target mRNAs and their diversity across cell types and contexts is not fully characterized, nor is it understood what 5′ UTRs features and structures confer dependency on DDX3X for proper translation regulation. Further, although DDX3X has been observed to interact with helix 16 of the 18S rRNA, a component of the small ribosomal subunit (Calviello et al. 2021), the function and extent of its association with the ribosome and other translation factors is still unclear. Given the substrate dependency of DDX3X's role in diseases, a better picture of such variables is necessary for insights into the broader landscape of translation regulation and its implications in health and disease.

In this paper, we created a helicase activity reporter for translation (HART) to measure the translational activity of DDX3X. We characterized the structures and features of 5′ UTRs who rely on DDX3X for proper translation and used HART to dissect this sensitivity to DDX3X. We then identified residues in the N terminus of DDX3X as mediating interaction with the ribosomal complex and used HART to determine that this interaction is important for DDX3X's role in promoting translation. Together, our work constitutes progress in the understanding of the mechanism of DDX3X-mediated translation and the creation of a versatile reporter with potential implications in both further dissections of DDX3X function and drug discovery.

## RESULTS

### Creating a reporter for the translational activity of DDX3X

**FIGURE 1.**
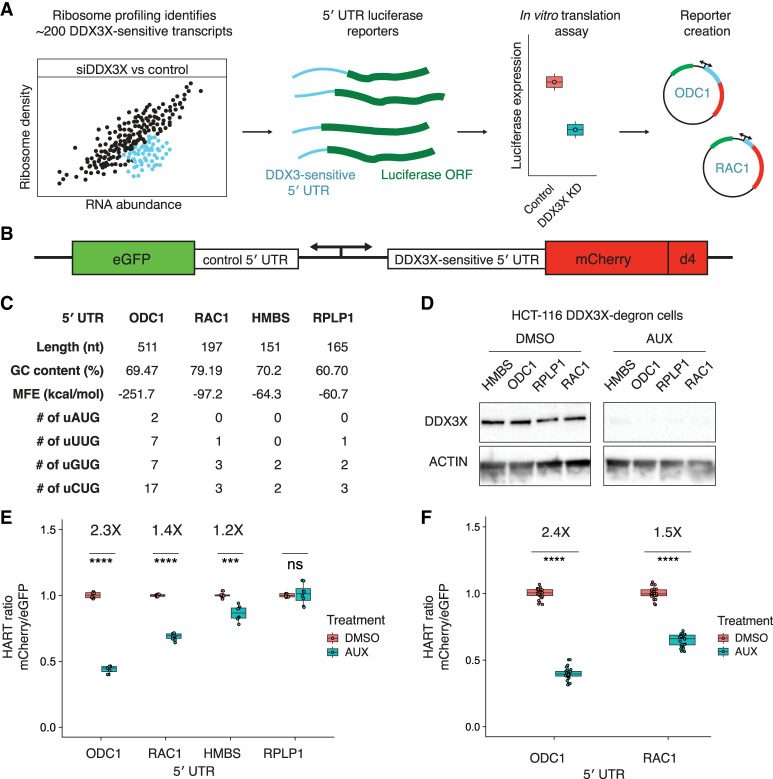
The HART uses DDX3X-sensitive 5′ UTRs to measure the translational activity of DDX3X. (*A*) Selection of DDX3X-sensitive 5′ UTRs for HART. Validated DDX3X-sensitive 5′ UTR from prior ribosome profiling and in vitro translation as well as negative controls were cloned into the HART reporter. (*B*) Diagram of HART. HART is constructed around a bidirectional promoter, with one arm directing the transcription of a control 5′ UTR and eGFP, and the other arm featuring a DDX3X-sensitive 5′ UTR followed by mCherry. An ornithine decarboxylase (ODC) degron (d4) shortens the mCherry half-life. The HART ratio (mCherry/eGFP) can be used to measure the translational activity of DDX3X. (*C*) Table of features for the 5′ UTRs from *C*. Features cataloged include length, GC content, RNA minimum free energy (MFE) prediction using the ViennaRNA Package (Lorenz et al. 2011), and the number of upstream AUGs and near-cognate codons. (*D*) Western blot for cells in *A*. HCT116 degron cells were treated with either auxin or DMSO, and then lysates were collected and resolved by SDS-PAGE and used for western blotting with antibodies against actin and DDX3X. (*E*) HART ratio in HCT116 degron cells analyzed with flow cytometry. HCT116 degron cells were lentivirally transduced with HART constructs with indicated 5′ UTRs upstream of mCherry. After 48 h from the addition of either DMSO or auxin, which induces degradation of endogenous DDX3X, the fluorescent signal of cells was measured by fluorescent cytometry. The HART ratio (mCherry/eGFP) was calculated for each cell and averaged across replicate wells. Data were obtained in three separate experiments for a total of nine replicates. Statistical significance was determined by unpaired *t*-test: (ns) *P* > 0.05, (*) *P* ≤ 0.05, (**) *P* ≤ 0.01, (***) *P* ≤ 0.001, (****) *P* ≤ 0.0001. (*F*) HART ratio in HCT116 degron cells analyzed by microscopy. HCT116 degron cells were transduced with HART construct for ODC1 or RAC1. After 48 h from the addition of either DMSO or auxin, the cells were fixed and imaged using an InCell Analyzer 6500HS. The HART ratio was calculated for each cell and averaged across replicate wells. Data were obtained in two separate experiments each for a total of 36 replicates. Statistical significance was determined by unpaired *t*-test: (ns) *P* > 0.05, (*) *P* ≤ 0.05, (**) *P* ≤ 0.01, (***) *P* ≤ 0.001, (****) *P* ≤ 0.0001.

We set out to create a reporter to investigate the translational activity of DDX3X. We first identified human transcripts whose translation is dependent on DDX3X. Ribosome profiling in prior work showed that the loss of DDX3X caused a decrease in ribosome density on a subset of transcripts, including ODC1, RAC1, and HMBS (Calviello et al. 2021). The 5′ UTRs of these transcripts require DDX3X for efficient translation in in vitro and in cell assays (Calviello et al. 2021; Wilkins et al. 2022). We define these sequences as DDX3X-sensitive 5′ UTRs and reasoned we could use them as the foundation of a reporter for DDX3X translation activity in cells (Fig. 1A,B).

We selected four 5′ UTRs—ODC1, RAC1, HMBS, and RPLP1—possessing differing features (Fig. 1C) and differing translational sensitivity to DDX3X (Calviello et al. 2021). We incorporated these 5′ UTRs into a construct we called HART (Fig. 1B). HART is a lentiviral plasmid featuring a dual promoter, composed of a copy of human PGK (hPGK) and one of mini CMV, which efficiently directs translation in a bidirectional fashion both in cells and in vivo (Amendola et al. 2005). We used hPGK to direct transcription of a DDX3X-sensitive 5′ UTR followed by the open reading frame (ORF) of the fluorescent protein mCherry. The mini CMV promoter was used to direct transcription of a DDX3X-insensitive 5′ UTR followed by the ORF of eGFP to act as an internal control. The DDX3X-insensitive 5′ UTR consists of a short 9 nt sequence (CTAGCCACC), which was previously used as a control in cellular and in vitro assays of DDX3X-sensitive translation (Calviello et al. 2021). To better detect changes in protein levels, we fused mCherry with the ODC degron (d4) known to confer a protein half-life of 4 h (Yen et al. 2008). In the HART reporter, mCherry protein levels are a proxy for the translation of DDX3X-sensitive transcripts, whereas eGFP protein levels are independent of DDX3X changes and act as an internal control. The mCherry/eGFP ratio, which we refer to as the HART ratio, is thus a readout of the ability of DDX3X to promote translation.

To validate HART as a reporter, we assessed the impact of DDX3X loss on the HART ratio. We depleted endogenous DDX3X using an inducible degron system, employing a male-derived colorectal cancer HCT 116 cell line with stably integrated auxin-activated OSTIR1 machinery, where the endogenous DDX3X gene was tagged with a degradation tag (Natsume et al. 2016; Calviello et al. 2021). Treating the cells with auxin for 48 h resulted in near complete degradation of endogenous DDX3X protein (Fig. 1D). We transduced HART into these degron cells and treated them with auxin for 48 h. Flow cytometry measurements of the ratio of mCherry to eGFP signal identified a significant decrease in auxin-treated cells compared to the DMSO-treated control in HART constructs containing HMBS, ODC1, or RAC1 but not RPLP1 5′ UTRs (Fig. 1E; Supplemental Fig. S1A,B). These results were in line with previous in cell and in vitro translation assays (Chen et al. 2015, 2016b; Calviello et al. 2021; Perfetto et al. 2021). Notably, there was no difference in eGFP fluorescence levels between auxin-treated cells and controls, whereas mCherry levels exhibited significant differences, demonstrating that the change in HART ratio between treatments was driven by the DDX3X-sensitive 5′ UTR (Supplemental Fig. S1C,D). To exclude an artifact due to the short control 5′ UTR, we also tested HART containing an 83 nt long 5′ UTR to direct translation of eGFP and found qualitatively similar results (Supplemental Fig. S1E,F). Microscopy measurements were consistent with flow cytometry, confirming the reliability and generalizability of HART-ODC1 and HART-RAC1 (Fig. 1F; Supplemental Fig. S1J,K). Overall, these results collectively demonstrated the effectiveness of HART as a reporter for evaluating DDX3X's impact on translation initiation.

### Structural probing of the RAC1 and ODC1 5′ UTRs

DDX3X is necessary for the proper translation of several human mRNAs, but the precise understanding of which 5′ UTR structures and features render a given transcript sensitive to DDX3X remains incomplete. Prior work has implicated upstream start codons, RNA structure, GC content, and other mRNA features as important in DDX3X-controlled regulation (Soto-Rifo et al. 2012; Chen et al. 2015; Lai et al. 2016; Calviello et al. 2021). However, experimental evidence of RNA structure in DDX3X-sensitive mRNAs is limited. To gain deeper insights into the elements that confer DDX3X sensitivity to the RAC1 and ODC1 5′ UTRs, we used SHAPE-MaP structure probing to experimentally determine their secondary RNA structures (Smola et al. 2015; Smola and Weeks 2018).

**FIGURE 2.**
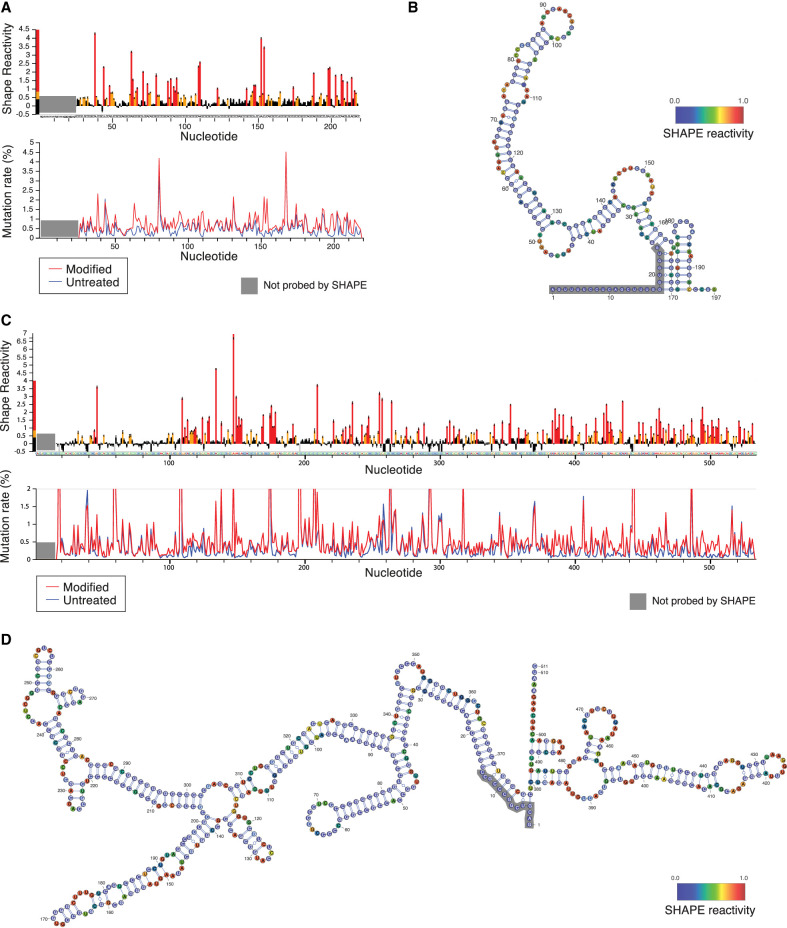
DDX3X-sensitive 5′ UTRs are highly structured. (*A*) SHAPE-MaP reactivity and mutation rate for the 5′ UTR of RAC1 in vitro. In vitro transcribed mRNA containing the 5′ UTR of RAC1 and the ORF of luciferase was probed with 200 mM NAI or DMSO control for SHAPE-MaP. The RNA was reverse transcribed, sequenced, and analyzed to obtain mutation profiles and SHAPE reactivity with the ShapeMapper tool. (*B*) Diagram of the structure of the RAC1 5′ UTR in vitro, based on data from *A* and computed with ShapeMapper 2.1.3 (Busan and Weeks 2018). (*C*) SHAPE-MaP reactivity and mutation rate for the 5′ UTR of RAC1 in vitro. In vitro transcribed mRNA containing the 5′ UTR of ODC1 and the ORF of luciferase was probed with 200 mM NAI or DMSO control for SHAPE-MaP. The RNA was reverse transcribed and sequenced. The mutation rate was calculated at each position for both treated and control samples. The SHAPE reactivity was calculated based on the difference in mutation rate. SHAPE reactivity is cropped for space, the full figure can be found in Supplemental Figure S2A. (*D*) Diagram of the structure of the ODC1 5′ UTR in vitro, based on data from *C* and computed with ShapeMapper 2.1.3 (Busan and Weeks 2018).

To measure RNA structures using SHAPE-MaP, mRNAs containing the RAC1 and ODC1 5′ UTRs and the luciferase ORF were in vitro transcribed using T7 polymerase (Calviello et al. 2021; Wilkins et al. 2022). RNA was then folded, probed with NAI or DMSO as control, and then reverse transcribed and sequenced. Mutation profiles and SHAPE reactivity were measured and used to model RNA secondary structures (Fig. 2A–D).

RNA structure prediction constrained by the SHAPE-MaP data for the RAC1 5′ UTRs predicts a complex structure, comprising a 152 nt long complex structure and a 23 nt stem–loop (Fig. 2A,B). The RAC1 5′ UTR has a 79% GC content and a predicted free energy of −97.20 kcal/mol. The RAC1 SHAPE-MaP result obtained in vitro is like the one obtained in cells (Supplemental Fig. S2B,C) or via in silico predictions using the RNAfold software (Supplemental Fig. S2D). The ODC1 5′ UTR also showed a highly complex structure, with a long 376 nt structure, with several smaller hairpins branching from it, and a shorter 107 nt structure, with an overall free energy of −251.70 kcal/mol (Fig. 2C,D; Supplemental Fig. S2A) and a 69% GC content. These in vitro probing results are overall similar to RNAfold prediction (Supplemental Fig. S2E).

The presence of large stem–loops and the overall high complexity of these 5′ UTRs could impede efficient translation initiation in the absence of the unwinding and remodeling activity of helicases (Pisareva et al. 2008). DDX3X may be crucial in resolving these stable stem–loops and enabling mRNA accommodating into the small ribosomal subunit or scanning across the 5′ UTR to identify the translation start site. Previous work has suggested that Ded1, the yeast homolog, has a higher unwinding activity than eIF4A, which may position Ded1/DDX3X as a specialist helicase required to unwind particularly complex structures (Gao et al. 2016).

### Dissection of RAC1 and ODC1 DDX3X sensitivity

**FIGURE 3.**
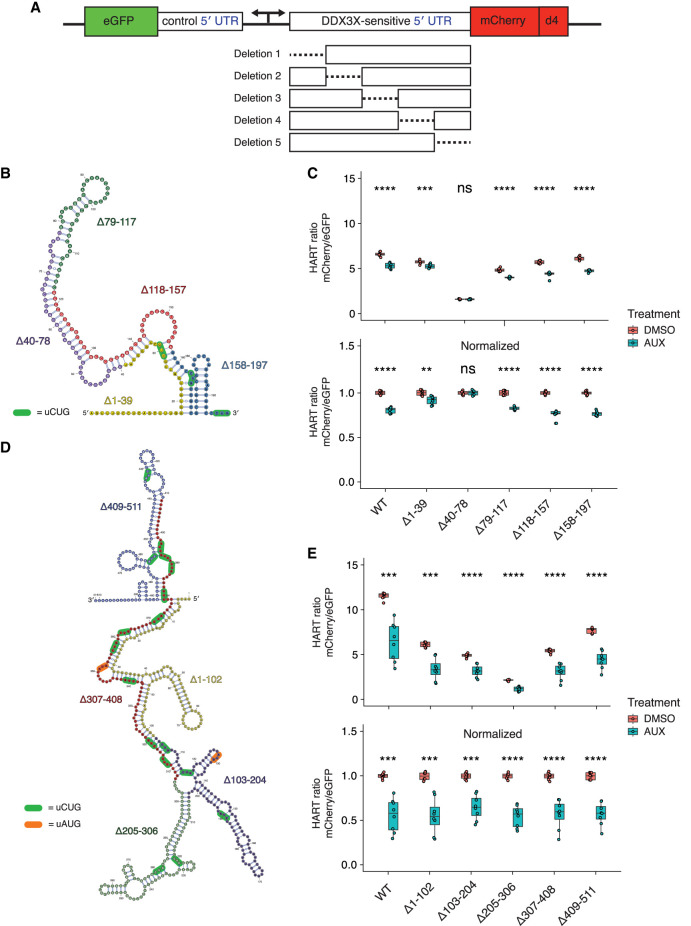
Dissection of RAC1 and ODC1 DDX3X sensitivity. (*A*) Schematic of the HART construct with deletions tiling DDX3X-sensitive 5′ UTRs. (*B*) Diagram of the structure of RAC1 with the deletions highlighted. (*C*) HART data for *B*. HART constructs containing RAC1 5′ UTR or five deletion mutants were transduced via lentivirus into HCT116 degron cells. The cells were treated with auxin to induce loss of endogenous DDX3X or DMSO control for 48 h, and the HART ratio (mCherry/eGFP) was measured by flow cytometry and presented as an absolute ratio (*upper* panel) or normalized to DMSO (*lower* panel). Data were obtained in three separate experiments for a total of nine replicates. Statistical significance was determined by unpaired *t*-test: (ns) *P* > 0.05, (*) *P* ≤ 0.05, (**) *P* ≤ 0.01, (***) *P* ≤ 0.001, (****) *P* ≤ 0.0001. (*D*) Diagram of the structure of ODC1 with the deletions highlighted. (*E*) Same experiment as in *C*, but with HART-ODC1 constructs from *D*. Data were obtained in three separate experiments for a total of nine replicates. Statistical significance was determined by unpaired *t*-test: (ns) *P* > 0.05, (*) *P* ≤ 0.05, (**) *P* ≤ 0.01, (***) *P* ≤ 0.001, (****) *P* ≤ 0.0001.

We next sought to determine which features of the ODC1 and RAC1 5′ UTRs contribute to DDX3X sensitivity. To explore this in an unbiased manner, we created HART constructs with five equal-size deletions tiling the 5′ UTRs of both RAC1 (Fig. 3A,B) and ODC1 (Fig. 3A,D). These constructs were then used to measure the HART ratio in HCT116 degron cells after 48 h of auxin or DMSO treatment.

Deletions of the 79–117, 118–157, and 158–197 regions of the RAC1 5′ UTRs residues conferred similar DDX3X sensitivity compared to full-length wild type. However, deletion of the 1–39 bases led to diminished sensitivity, whereas deletion of the 40–78 bases showed an even further reduction to the point at which there was no statistical difference between cells treated with auxin or DMSO. The 1–39 and 40–78 deletions impact the large structure in the RAC1 5′ UTR, suggesting that unwinding of this structure by DDX3X is important in the scanning of its 5′ UTRs (Fig. 3B). This is consistent with prior work that identified an important role for cap-proximal RNA structures in DDX3X-mediated translation (Soto-Rifo et al. 2012). Notably, in silico folding predicts the Δ40–78 RAC1 5′ UTRs possess the lowest free energy among all the deletions, suggesting that a less stable structure exhibits decreased sensitivity to DDX3X (Supplemental Fig. S3). In contrast, we find that deletions in the ODC1 5′ UTR do not impact DDX3X sensitivity, suggesting its overall GC content or structure may be sufficiently complex that no single deletion ablates a requirement for DDX3X (Fig. 3E). Taken together, we find that DDX3X sensitivity is mediated by regions within some but not all DDX3X-sensitive mRNAs.

### The 38–44 residues of DDX3X contribute to its association with the translational machinery

The dynamics of DDX3X unwinding of 5′ UTR structures during ribosome accommodation and/or scanning are currently unknown. One possibility is that DDX3X unwinds structures ahead of ribosome scanning, independently and untethered from the ribosome or other ribosome-associated factors in a *trans* fashion. Alternatively, it is possible that DDX3X is bound to either the ribosome or the translational machinery during ribosome scanning and that it unwinds 5′ UTRs while tethered, in a *cis* fashion. The *cis* scenario would be like the behavior of related DEAD-box RNA helicase eIF4A that associates with the ribosome during scanning (Brito Querido et al. 2020). Some evidence for the *cis* model is provided by PAR-CLIP data, which suggests that DDX3X binds to the helix 16 of the 18S rRNA in the small subunit of the ribosome, which is located near the mRNA entry channel (Calviello et al. 2021). Interestingly, a similar result was also found in yeast, where iCLIP identified helix 16 as cross-linking with Ded1, the yeast homolog of DDX3X (Guenther et al. 2018).

**FIGURE 4.**
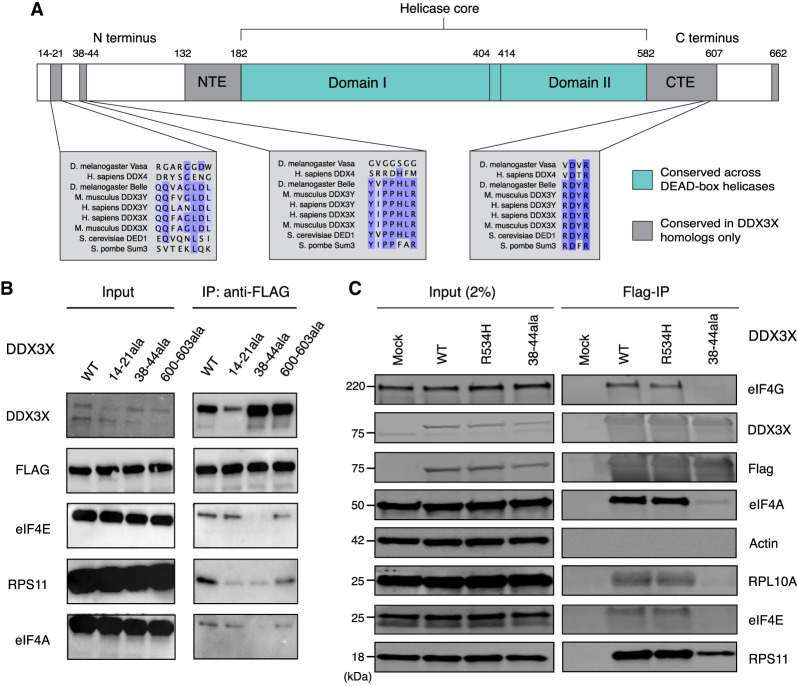
DDX3X interacts with the translational machinery via the 38–44 residues. (*A*) Diagram of the DDX3X protein structure. DDX3X contains a helicase core, highly conserved across the DEAD-box helicase protein family, composed of two RecA-like domains, denoted as Domain I and Domain II (denoted in blue). In addition to the helicase core, the functional core of the protein also includes the NTE and CTE, which have been shown to be necessary for the RNA unwinding activity of DDX3X. Outside of the functional core, there are the N and C termini, which are less conserved across the protein family, but contain regions conserved across the Ded1/DDX3X subfamily (denoted in gray). (*B*) Immunoprecipitation of DDX3X mutants. HEK 293T cells were lentivirally transduced with FLAG-tagged DDX3X WT or several mutants, including the helicase defective mutant R534H and three N- and C-termini mutations in sites conserved across the DDX3X/Ded1 subfamily. Immunoprecipitation was conducted for FLAG and run on western blot, staining for ribosome-related proteins and controls. Note that for DDX3X 14–21, the DDX3X antibody signal is lowered because the DDX3X epitope is affected by the 14–21 deletion. (*C*) DDX3X coimmunoprecipitation with translation machinery proteins. HEK 293T cells were transduced with FLAG-tagged DDX3X WT, R534H, or 38–44ala. Immunoprecipitation was conducted for FLAG and immunoblotted for ribosome-related proteins and controls.

Although helix 16 is a potential location of the binding of DDX3X to the ribosome, it is unknown what part of the DDX3X protein would be responsible for this putative interaction. DDX3X is constituted by two RecA-like domains (Domain I and Domain II) that make up the helicase core, which is highly conserved across the DEAD-box helicase family and across species. The helicase core is flanked by the N`-terminal extension (NTE) and the C-terminal extension (CTE), which were found to also be essential to the protein's functional helicase activity (Epling et al. 2015; Floor et al. 2016). Finally, the N terminus and the C terminus are present at the extremities of the protein and are significantly less conserved across the protein family and across species (Fig. 4A), although they contain several regions conserved across the DDX3X/Ded1 subfamily (Sharma and Jankowsky 2014).

DDX3X could interact with the translational machinery either through the functional core, one or both termini, or multiple locations. One factor that suggests this interaction is happening in the termini is the possible conservation of this binding between DDX3X and its yeast homolog Ded1 (Guenther et al. 2018). Taken together, these data suggest that the N and C termini of DDX3X play a functional role and may potentially mediate interaction with the ribosome or its associated factors.

Having identified the N and C termini as locations potentially mediating binding to the translational machinery, we then attempted to narrow down the location of the binding domain. We made constructs of FLAG-tagged DDX3X with alanine substitutions across termini residues conserved across the DDX3X family, including amino acids 14–21, 38–44, and 600–603 (Fig. 4B). These constructs were transduced into HEK 293T cells from which RNase-treated lysates were obtained and used for FLAG immunoprecipitation. DDX3X WT pulled down ribosomal subunit proteins RPS11 and RPL10A as well as translation factors eIF4E, eIF4G, and eIF4A, which are associated with the scanning ribosome (Kumar et al. 2016). DDX3X R534H, a missense mutant found in the functional core, which causes helicase activity deficiency (Floor et al. 2016; Calviello et al. 2021), showed a similar coprecipitation profile as wild type. Conversely, the different mutants lost interaction with these proteins to different degrees. Both 14–21 and 600–603 mutants showed a similar ability to pull down eIF4E, eIF4G, and eIF4A, but a reduced ability to pull down RPS11. Finally, the mutant with alanine substitutions spanning the 38–44 residues, which we refer to as 38–44ala, presented the most severe difference from wild type, with greatly reduced copurification of RPS11, eIF4E, eIF4G, and eIF4A (Fig. 4B,C). This suggests that the 38–44 residues of DDX3X are involved in the interaction with the ribosome, whether directly or indirectly through other intermediary proteins.

The 38–44 residues (YIPPHLR) are conserved across the DDX3X/Ded1 subfamily but absent in other DEAD-box proteins (Fig. 4A; Sharma and Jankowsky 2014). This sequence has been previously identified as potential eIF4E-binding region because it is reminiscent of the consensus eIF4E-binding sequence YXXXXLΦ found in several eIF4E-binding proteins such as the 4EBPs and eIF4G (Supplemental Fig. S4A). Substitution of Tyr38 or Leu43 to alanine partially ablated coimmunoprecipitation of DDX3X and eIF4E in HeLa lysate (Shih et al. 2008), in a fashion similar to our observation of the lack of coprecipitation between 38–44ala and eIF4E. We hence decided to test whether these residues mediate DDX3X's indirect interaction with ribosome via direct binding to eIF4E.

We used NMR spectroscopy to test whether the 38–44 region of DDX3X interacts with eIF4E directly. We purified ^15^N labeled *Saccharomyces cerevisiae* eIF4E, which shares the core conserved peptide binding site with human eIF4E (Joshi et al. 2004). The ^15^N HSQC spectrum of eIF4E bound to m^7^GTP was well-dispersed, indicating a stable, folded protein (Supplemental Fig. S4B). The addition of 1 mM of a peptide comprising human 4E-BP1 amino acids 51–67 elicited dramatic chemical shift changes (Supplemental Fig. S4C), consistent with the known interaction between 4E-BP1 and eIF4E. In contrast, the addition of a peptide comprising human DDX3X amino acids 33–51 caused minimal chemical shift perturbations (Supplemental Fig. S4D). We, therefore, conclude that human 4E-BP1 binds to *S. cerevisiae* eIF4E, but that DDX3X amino acids 33–51 do not directly interact with eIF4E.

The lack of coimmunoprecipitation between mutant DDX3X and ribosomal proteins could be due to mutants affecting protein localization. To test this possibility, we used microscopy to determine the localization of the different DDX3X variants. HEK 293T cells were transduced with different DDX3X-FLAG constructs, fixed, and stained for anti-FLAG and the nuclear marker DRAQ7. DDX3X protein was broadly cytoplasmic, and neither the R534H mutation, nor 38–44ala, nor the double mutant 38–44ala/R534H altered this localization compared to WT (Supplemental Fig. S4E).

We have, therefore, identified the 38–44ala residues as contributors to the interaction between DDX3X and the ribosome complex.

### Translation complex association plays a role in the DDX3X function

Having identified a DDX3X mutant that impacts its association with the ribosome, we next investigated the effect of losing this interaction on DDX3X's translational activity. HCT116 degron cells were transduced with both the HART construct and DDX3X-FLAG-BFP, either WT or helicase defective R534H, 38–44ala, or the double mutant 38–44ala/R534H. FACS was used to obtain a triple-positive population for mCherry, eGFP, and BFP. These cells were treated with auxin for 48 h, and flow cytometry was used to measure the HART ratio.

**FIGURE 5.**
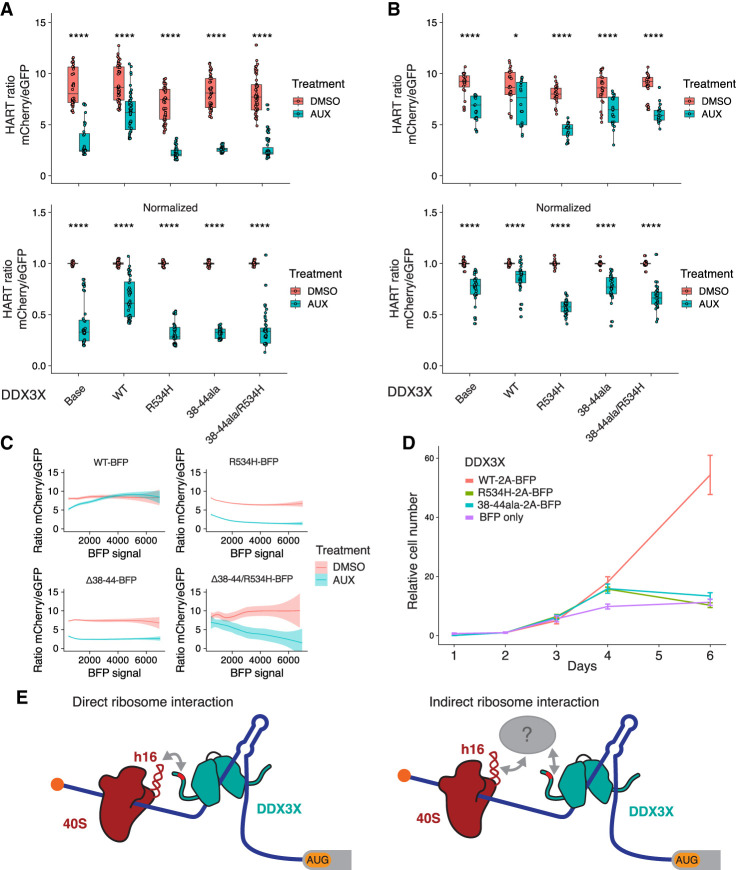
DDX3X 38–44ala cannot rescue translational or cell growth defects caused by the loss of DDX3X. (*A*,*B*) HART data for indicated DDX3X mutants. HCT116 degron cells were transduced with the HART-ODC1 (*A*) or HART-RAC1 (*B*) construct and different variants of DDX3X-FLAG-BFP, either WT, helicase defective R534H, 38–44ala, or the double mutant R534H/38–44ala. Cells were treated with auxin, which induces loss of the endogenous DDX3X via a degron tag, or DMSO as control. After 48 h flow cytometry was used to measure fluorescence levels, and the HART ratio (mCherry/eGFP) was calculated and presented as an absolute ratio (*upper* panel) or normalized to DMSO (*lower* panel). Data were obtained in three separate experiments for a total of 18 replicates. Statistical significance was determined by unpaired *t*-test: (ns) *P* > 0.05, (*) *P* ≤ 0.05, (**) *P* ≤ 0.01, (***) *P* ≤ 0.001, (****) *P* ≤ 0.0001. (*C*) Relationship between DDX3X-FLAG-BFP levels and HART ratio. Flow cytometry was used to measure eGFP and mCherry levels, which constitute the HART ratio, and BFP, which is a proxy for the level of DDX3X-FLAG-BFP expression. (*D*) Cell growth curve for DDX3X variants. HCT116 degron cells were transduced with DDX3X WT, R534H, 38–44ala, or BFP control. After auxin was added to degrade endogenous DDX3X, CellTiter-Glo was used to measure cell number and plot cell growth. Data were obtained in two separate experiments for a total of eight replicates. (*E*) Model of DDX3X function in translation and its relation to the ribosome. The 38–44 residues of DDX3X (shown in red) contribute to its interaction with the translational machinery. Helix 16 has been previously identified as mediating this association, but it is unknown whether this interaction happens directly between DDX3X and the ribosome (*left*) or via intermediary protein(s) such as the components of the eIF4F complex (shown in gray). Our data show that the association between DDX3X and the translational machinery plays a crucial role in DDX3X's role of unwinding 5′ UTRs to allow ribosome scanning and promote translation of a subset of human mRNAs.

The HART-ODC1 ratio of auxin-treated cells cotransduced with WT DDX3X was increased compared to parental cells, although not to the same level as control DMSO-treated cells. This suggests that the exogenous DDX3X partially rescued the translation defect caused by the loss of endogenous DDX3X. Instead, transduction of R534H nor 38–44ala nor the double mutant 38–44ala/R534H did not rescue the effect (Fig. 5A; Supplemental Fig. S5A,C). Similarly, results for HART-RAC1 showed that transduced WT DDX3X, but not R534H, 38–44ala, nor R534H/38–44ala DDX3X, can partially rescue loss of endogenous DDX3X (Fig. 5B; Supplemental Fig. S5B). The inability of DDX3X 38–44ala to rescue the phenotype suggests that the interaction between DDX3X and the translational machinery is necessary for DDX3X's role during ribosome scanning, in line with the *cis* model of DDX3X function. Notably, the addition of exogenous DDX3X R534H, even in the presence of endogenous WT protein in the DMSO condition, decreased the HART ratio, in line with the dominant negative effect of R534H and similar missense mutations seen in clinical data (Epling et al. 2015; Lennox et al. 2020; Calviello et al. 2021; Gadek et al. 2023).

Lentiviral transduction can lead to variable expression of the exogenous construct across cells. We used the BFP signal, which is a proxy for the expression of DDX3X-FLAG, to investigate the effect of DDX3X expression level on the HART ratio. Notably, higher BFP levels in DDX3X-FLAG-BFP-expressing cells correlated with a reduction in the difference of the HART ratio between auxin and DMSO-treated cells, indicating dosage sensitivity (Fig. 5C). At high levels of BFP, the ratio in auxin-treated cells was above those in DMSO-treated cells, suggesting that high enough levels of exogenous DDX3X could promote translation above endogenous levels. This phenomenon was not observed in cells transduced with the mutant DDX3X constructs.

We also investigated the effect of DDX3X mutants on cellular growth. Although exogenous DDX3X WT rescued cell growth defects due to the loss of endogenous DDX3X, neither mutant R534H nor 38–44ala can (Fig. 5D). Similarly, the 132–607 truncation of DDX3X, which lacks N and C termini but preserves helicase activity (Floor et al. 2016), cannot rescue this cell growth phenotype (Supplemental Fig. S5A). These data suggest that both helicase function and the association with the translational machinery are crucial to DDX3X function in proliferation, although the exact interactions are yet to be determined.

Our data show that the 38–44 residues of DDX3X contribute to its interaction with the translational machinery. This interaction, whether direct with the ribosome or mediated by other factors, is important for DDX3X's role in unwinding secondary structures in the 5′ UTR of a subset of human mRNAs and promoting their translation (Fig. 5E).

## DISCUSSION

In this paper, we investigated the translational activity of DDX3X. DDX3X-sensitive 5′ UTRs were selected among transcripts whose ribosome density went down upon loss of DDX3X and whose 5′ UTR was shown to be sensitive to DDX3X (Calviello et al. 2021). We chose the ODC1 and RAC1 5′ UTRs as they were among the most DDX3X-sensitive transcripts in both in vitro and *in cell* assays. They also provide diversity in length, at 511 and 197 nt, respectively. We used the ODC1 and RAC1 5′ UTRs to create a DDX3X translation reporter, termed HART. Notably, HART data replicated the previous assays, with the translation of ODC1 and RAC1 5′ UTRs shown to be sensitive to the loss of DDX3X, unlike the RPLP1 5′ UTR (Calviello et al. 2021). Being embedded into the cell as DNA via lentiviral transduction, reporter-encoded mRNAs undergo mRNA processing and export before they are translated. The alignment between HART data and prior in vitro and in cell assays, which are performed with in vitro transcribed and capped mRNA, suggest that the DDX3X-sensitive step is in translation. Differential DDX3X dependence across 5′ UTRs attached to the same coding sequence and 3′ UTR isolates DDX3X dependence to a step before translation elongation.

To promote the translation of eGFP in HART and act as an internal control, we selected a short 9 nt 5′ UTR, which is too short to form complex RNA structures that might depend on DDX3X for unwinding before translation. Additionally, the lack of change of eGFP signal between auxin and DMSO conditions in HART (Supplemental Fig. S1C) suggests that the loss of DDX3X does not affect its translation. In addition to its lack of complex RNA structure, there could be other reasons that make it insensitive to permutations in DDX3X. Very short 5′ UTRs promote translation in cap-dependent and -independent mechanisms (Trainor and Shcherbik 2021). Additionally, even in the context of cap-dependent translation, they have been observed to direct translation in a ribosome scanning–free manner (Elfakess et al. 2011; Haimov et al. 2015). For example, the translation initiator of short 5′ UTR (TISU) is a regulatory element that has a median length of 12 nt and is required for a cap-dependent, scanning-free mechanism of translation initiation on 5′ UTRs with fewer than 30 nt (Elfakess et al. 2011). Notably, the TISU function does not require eIF4A, suggesting that some short 5′ UTRs lack the need for DEAD-box helicases (Sinvani et al. 2015). Similarly, cap-assisted translation without scanning was reported for the histone H4 mRNA, which is notable for directing translation in a wide variety of eukaryotic cells (Dasmahapatra et al. 1987; Martin et al. 2011; Trainor and Shcherbik 2021). It is possible that this extremely short 5′ UTR avoids DDX3X dependency by initiating translation without ribosome scanning or by cap-independent mechanisms altogether, hence without the need for the unwinding of complex structures. Further work into the mechanism of translation of RNAs with very short 5′ UTR is still needed to understand this phenomenon. To exclude that the HART data are an artifact of using a short 5′ UTR, we replicated our results using an 83 nt long control 5′ UTR to promote eGFP expression (Supplemental Fig. S1E,F). Taken together, and in conjunction with HART's reproducibility in microscopy (Fig. 1F), the data suggest that HART is a reliable and versatile reporter for the activity of DDX3X in translation initiation.

One crucial yet incompletely understood aspect of DDX3X regulation is what structures and features make a 5′ UTR translationally DDX3X-sensitive. We used HART to dissect the features of DDX3X-sensitive 5′ UTRs. Both the RAC1 and ODC1 5′ UTRs have high GC content and are predicted by SHAPE-MaP to possess complex secondary structures. This is in line with previous data suggesting high GC content as a marker of DDX3X sensitivity (Oh et al. 2016; Calviello et al. 2021) as well as data in yeast, where repression of Ded1p activity leads to accumulation of organized RNA structures in 5′ UTRs (Guenther et al. 2018). Elaborate RNA secondary and tertiary structures can sterically block the scanning ribosome, which possesses no intrinsic unwinding activity and relies on helicases such as DDX3X (Leppek et al. 2018).

Our work suggests that the RAC1 5′ UTR features a large stem–loop which relies on DDX3X for unwinding. In the absence of DDX3X, such structure impedes the ribosome's ability to scan and initiate elongation. Disruption of this structure with deletions of nucleotides 1–39 and 40–78 decreases sensitivity to DDX3X (Fig. 3B) and produces 5′ UTRs with lower predicted free energy and structural complexity. Strikingly, the 40–78 deletion causes the reporter to be unaffected by DDX3X loss. Another possibility is that this stem–loop regulates uORF usage. Secondary structures in the 5′ UTRs can also increase ribosome dwelling time and promote translation on suboptimal upstream start codons (Kozak 1991; Hinnebusch et al. 2016). The RAC1 5′ UTRs feature a CUG near-cognate start codon in position 28, right upstream of the stem–loop (Fig. 3B). In the absence of DDX3X, the stem–loop might direct translation toward the uCUG, which might negatively affect translation of the main ORF. This model is consistent with the diminished dependence of DDX3X in the 1–39 and 40–78 deletion constructs. Overall, the data suggest that sensitivity to DDX3X in RAC1 and ODC1 5′ UTRs is conferred by complex secondary structures.

We envision two models of DDX3X unwinding 5′ UTRs structures during ribosome scanning. In the *trans* model, DDX3X unwinds structures ahead of and untethered from the scanning ribosome and associated translational machinery. In the *cis* model, DDX3X unwinds the RNA while tethered or bound, similarly to how eIF4A associates with the ribosome during scanning (Brito Querido et al. 2020). To differentiate between these two models, we investigated the potential binding of DDX3X to the translational machinery. We hypothesized an interaction between the terminal domains of DDX3X and the ribosome or its associated factors. Notably, these termini are highly conserved from yeast to human across the Ded1/DDX3X subfamily, but not in the wider DEAD-box family (Sharma and Jankowsky 2014; Floor et al. 2016). Both DDX3X and Ded1 have been suggested to bind to helix 16 of the 18S of the smaller subunit of the ribosome (Guenther et al. 2018; Calviello et al. 2021). We reasoned that if this interaction with the ribosome or associated factors is specific to the subfamily, the residues mediating it are likely to be found in the termini.

To determine which residues in DDX3X mediate its interaction with the translation machinery, we made targeted alanine substitutions to several conserved regions in DDX3X termini. Immunoprecipitation showed the 38–44ala mutation, but not DDX3X WT and R534H, lost interaction with ribosomal protein RPS11 and several translation initiation factors (Fig. 4C). Although it was previously identified as an eIF4E-binding region, NMR data showed that the 38–44 residues of DDX3X did not bind directly to eIF4E (Supplemental Fig. S4D). This can be explained by the fact that the 38–44 region of DDX3X is similar to the canonical eIF4E-binding region found in 4EBP and eIF4G but differs in its last residue, which is a hydrophobic amino acid in the canonical sequence but an arginine in DDX3X (reviewed by Sachs and Varani 2000; von der Haar et al. 2004; Richter and Sonenberg 2005). Residues 38–44 could instead mediate binding to the translational machinery via other factors. Previous work has also suggested that DDX3X interacts with eIF4G (Soto-Rifo et al. 2012; Soto-Rifo and Ohlmann 2013; Ayalew et al. 2016; Gong et al. 2021). Direct binding to yeast eIF4G, 4A, and 4E was also seen in Ded1 (Hilliker et al. 2011; Guenther et al. 2018; Gulay et al. 2020), suggesting interaction between DDX3X and the eIF4F complex (Ryan and Schröder 2022). Notably, deletion of the entire N-terminal region of DDX3X had a minor effect on RNA helicase activity, indicating that the 38–44 truncation should have at most a minor effect on enzymatic activity (Floor et al. 2016). We suggest that the 38–44 residues of DDX3X contribute, at least partially, to the interaction with the ribosome, although whether this interaction is direct or mediated by other factors in the translational machinery needs further study (Fig. 5E). Data obtained with HART in conjunction with the DDX3X 38–44ala mutant showed decreased translation of DDX3X-sensitive transcripts, underscoring how the DDX3X-translational complex interaction is necessary for DDX3X's role in translation.

In addition to its role in dissecting specific 5′ UTRs sensitivities and DDX3X mutant effects, we anticipate HART to be useful in several other applications. Its reliability as a 5′ UTR–based reporter of function in translation initiation can be used in conjunction with large naturally occurring or artificial 5′ UTR libraries to gain a granular and large-scale characterization of DDX3X regulation. Alternatively, HART's readout in both flow cytometry and microscopy settings can be used to screen for drugs that alter DDX3X behavior or rescue DDX3X or mutation. The lentiviral transduction delivery of HART can be applied to in vivo systems, in order to measure DDX3X function in living systems. Finally, by choosing the proper control and experimental 5′ UTRs, it can be adapted to measure the activity of any other helicase or translation factor in addition to DDX3X. HART's reliability and flexibility can prove a useful tool to study translation in diverse settings and advance both the understanding of protein production and disease treatment.

## MATERIALS AND METHODS

### Molecular cloning

DNA amplification was obtained by PCR with either KAPA HiFi HotStart ReadyMix (Roche KK2601) or Q5 High-Fidelity 2× Master Mix (New England Biosystems M0492) following manufacturer protocol unless otherwise noted. PCR products were digested with 1 μL DpnI (NEB R0176L) for 1 h and purified by agarose gel extraction (MinElute Gel Extraction Kit, QIAGEN 28606). Plasmid constructs were created by Gibson cloning with NEBuilder HiFi DNA Assembly Master Mix (NEB E2621L) or Gibson Assembly Master Mix (NEB E2611L). Assembled plasmids were transformed into Mach1 (Invitrogen C862003) or STBL3 (from Q3 Macrolab at the University of California) competent cells for amplification and were purified with QIAprep miniprep (QIAGEN 27104).

### DNA sequences

The lentiviral dual promoter plasmid backbone for HART was a gift of the Goodarzi laboraotry and published in Amendola et al. (2005). The 5′ UTRs of RAC1, HMBS, ODC1, and RPLP1 and control were taken from plasmids from previous work (Calviello et al. 2021). Deletions in RAC1 and ODC1 were achieved by Gibson cloning. The mutated d4 ODC degron fused to mCherry was inserted via primers with Gibson cloning based on the published sequence: (AGCCATGGCTTCCCGCCGGAGGTGGAGGAGCAGGATGATGGCGCGCTGCCCATGTCTTGTGCCCAGGAGAGCGGGATGGACCGTCACCCTGCAGCCTGTGCTTCTGCTAGGATCAATGTG) (Yen et al. 2008). Gibson cloning was used to insert all the HART elements into the lentivirus UCOE-SFFV backbone. The final HART construct is:

PuroR-T24-eGFP-[control5′ UTR]-miniCMV-hPGK-[DDX3X- sensitive5′ UTR]-mCherry-d4ODCdegron.

Plasmids were deposited on Addgene:

    #207398: KW54 (HART-ODC1)
    #207399: KW57 (HART-RAC1)
    #207400: KW63 (HART-RAC1-PuroR)
    #207401: KW78 (HART-ODC1-PuroR)

The 83 nt control 5′ UTR used in Supplemental Figure S1E,F has the sequence TCAGATCGCCTGGAGACGCCATCCACGCTGTTTTGACCTCCATAGAAGACACCGGGACCGATCCAGCCTCCGCGGAATTCACC and was obtained from Amendola et al. (2005).

Lentiviral DDX3X constructs were created by inserting the DDX3X sequence from previous work (Calviello et al. 2021) into the UCOE-SFFV backbone (gift from the James K Nunez laboratory) via Gibson cloning. Lentivirus packaging plasmids included gag/pol, REV, TAT, and VSVG (gift from the Weissman laboratory). Gibson cloning was also used to generate the various mutations via primers containing said mutations. BFP and puromycin resistance sequences were taken from pHR-SFFV-dCas9-BFP-KRAB (Addgene 46911) and Twist, respectively. The final DDX3X lentiviral construct sequence is: UCOE-SSFV-DDX3X-3xFLAG-2xP2A-TagBFP-PuromycinR.

### Cell culture

HCT116 cells were cultured in McCoy's 5A media (Gibco 16600082) and 10% FBS (Avantor 97068-085) and 1× Penicillin–Streptomycin Solution (Corning 30002CI). HEK 293T cells were cultured in DMEM with 4.5 g/L glucose, l-glutamine, and sodium pyruvate (Corning 10-0130CV) and 10% FBS (Avantor 97068-085) and 1× Penicillin–Streptomycin Solution (Corning 30002CI). Cells were cultured at 37°C in 5% CO_2_. Cell passage was conducted with trypsin (Corning 25-053-CI). Cells were washed with 1× PBS pH 7.4 (Gibco 70011044) before media change and passaging.

### Lentivirus packaging and infection

For packaging lentivirus, HEK 293T cells were kept at confluency below 90% for several passages. On day 1, HEK 293T cells were plated at 1.2 × 10^6^ cells/well in 6 well plates. On day 2, cells were transfected with lentiviral DNA following the Mirus-LT1 (Mirus MIR 2300) transfection protocol. Briefly, 6 μL transfection reagent Mirus-LT1 was mixed with 250 μL Opti-MEM (Thermo Fisher 31985062) and incubated at RT for 15 min. 1.5 μg of the desired lentiviral DNA with 0.1 μg gag/pol, REV, and TAT packaging plasmids and 0.2 μg VSVG packaging plasmid (for a total of 2 μg of DNA) were added to the Opti-MEM mix. The mix was incubated at RT for 15 min, and then gently added to the HEK 293T cells dropwise. On day 2, the virus-containing supernatant was harvested by gentle pipetting and filtered with a low protein binding syringe filter (0.22 μM, EDM Millipore SLGV033RB). The virus was frozen at −80°C for long-term storage or transduced directly into the desired cells.

Cells to be transduced were plated in 24 well plates at 0.168 × 10^6^ cells/well on day 1. On day 2, the lentivirus supernatant collected previously was added to the cells. Different amounts of virus, ranging from 5 to 500 μL, were added together to Polybrene (EMD Millipore TR-1003-G) to each cell. The plates were spun down for 2 h at 1000 RPM at 37°C. On day 4, cells were sorted as described below. The appropriate amount of virus was determined by sorting cells from wells with infection rates below 30%.

Cells in Figures 1 and 3 were transduced with HART constructs containing the indicated DDX3X-sensitive 5′ UTR. Cells in Figure 4 were transduced with DDX3X constructs. Cells in Figure 5 were transduced with both the indicated HART and DDX3X constructs.

### Flow cytometry

For cells transduced with the HART constructs in Figures 1 and 3, FACS was used to sort cells positive for both eGFP and mCherry. For cells transduced with the DDX3X constructs in Figure 4, cells positive for BFP were sorted. For cells transduced with both HART and DDX3X constructs in Figure 5, cells positive for eGFP, mCherry, and BFP were sorted.

FACS was performed using Fortessa SORP to measure the fluorescent signal of eGFP (488 nm 50 mW laser, Blue B or Blue C), mCherry (561 nm 50 mW laser, YG C detector), and BFP (405 nm 100 mW laser, Violet F detector). Untransduced cells were used as negative controls for gating after filtering for single cells.

Sorted cells were used for the HART experiments in Figures 1, 3, and 5. Cells were plated in 24 well plates at 0.168 × 10^6^ cells/well with 2 mL of media. 500 mM Auxin (indole-3-acetic acid) in DMSO (Corning 25950CQC) or DMSO alone was added at a 1:1000 dilution to the media. After 48 h, cells were trypsinized with 200 μL trypsin and resuspended with 200 μL media. Cells were then flowed with a Fortessa X20 Dual SORP. Untransduced cells were used as negative controls for gating after filtering for single cells. Cells were gated as single cells and double positive for eGFP (488 nm 60 mW laser, Blue C detector) and mCherry (561 nm 50 mW laser, YG C detector) for Figures 1 and 3, and as triple negative for eGFP, mCherry, and BFP 405 nm 100 mW laser, Violet F detector) in Figure 5. Each experiment used 4 wells per condition, and each well was measured twice. The raw value of the three channels was exported. The ratio of mCherry/eGFP was calculated as the HART readout. Data were normalized by dividing the value of each replicate, both DMSO- and auxin-treated wells, by the value of the DMSO-treated wells.

### Cell growth curves

Cells were plated in opaque 96 well plates at 5000 cells per well. Cell number was measured at the indicated time intervals with the CellTiter-Glo Luminescent Cell Viability Assay (Promega G7571) following the manufacturer's instructions.

### RNA folding prediction

RNA folding predictions were computed using the ViennaRNA Package (Version 2.5.1) (Zuker and Stiegler 1981; Lorenz et al. 2011).

### SHAPE-MaP

SHAPE-MaP was conducted as described (Smola et al. 2015; Smola and Weeks 2018). First, we in vitro transcribed RNA as described (Wilkins et al. 2022). RAC1 and ODC1 5′ UTRs were placed under the control of a T7 promoter and in front of luciferase ORF and transcribed using T7 polymerase (Calviello et al. 2021; Wilkins et al. 2022). For Shape-MaP, 1000 ng of the transcribed RNA in 12 μL water was denatured at 95°C for 2 min and then snap cooled on ice for 2 min. Six microliters of the 3.3× folding buffer (333 mM HEPES pH 8.0, 333 mM NaCl, 33 mM MgCl_2_) were added to each sample and the RNA was folded at 37°C for 20 min. For the unfolded control, 1000 ng RNA was placed in 10 μL formamide, 2 μL 10× DC buffer (500 mM HEPES [pH 8.0], 40 mM EDTA), and 6 μL water and denatured at 95°C for 2 min.

For RNA modification, 1 μL of either DMSO or 2M NAI was added to the RNA and mixed. The folded RNA was incubated 15 min at 37°C, while the unfolded RNA was incubated 15 min at 60°C. Two microliters of 100 mM DTT was added to all tubes to quench the reaction. Water was added to a volume of 50 μL and the RNA was cleaned up (Zymo RNA clean and concentrator -5 R1013) and eluted in 15 μL water.

For reverse transcription, 1.5 μL 200 ng/μL of random reverse transcription primers was added to the 15 μL RNA tubes and the reaction was incubated at 65°C for 10 min and then at 4°C for 2 min. Fresh MaP buffer was prepared by mixing equal volumes 5× MaP prebuffer (250 mM Tris [pH 8.0], 375 mM KCl, 50 mM DTT, 2.5 mM dNTP each) and 30 mM MnCl_2_. Twelve microliters of 2.5× MaP buffer was added to the RNA tubes and mixed and incubated at 25°C for 2 min. One microliter of Superscript II reverse transcriptase (Thermo Fisher Scientific 18064014) was added and incubated at 25°C for 10 min, then at 42°C for 90 min, then 10 cycles of 50°C 2 min and 42°C 2 min, and then 70°C for 10 min to deactivate the reverse transcriptase. The reactions were placed on ice and then purified using Zymo DNA clean and concentrator kit -5 (Zymo D4004) following cDNA protocol. The cDNA was then amplified by PCR and sequenced using Amplicon-EZ (Azenta Lifesciences). ShapeMapper and SuperFold packaged were used to analyze the data, convert it into mutational profiles, create SHAPE reactivity plots, and model RNA secondary structures (Busan and Weeks 2018). VARNA software was used for the automated drawing, visualization, and annotation of the secondary structures (Darty et al. 2009).

For the *in cell* SHAPE-MaP, HART-ODC1 and HART-RAC1 cells were grown to ∼80% confluency in 24 well plates. Cells were washed with PBS. One hundred and fifty microliters of 300 mM NAI in PBS was added to each well and the same volume of DMSO in PBS was added to control cells. The reaction was incubated at 37°C for 30 min. Two hundred microliters of quench solution (700 mM 2-mercaptoethanol in 1× PBS, made immediately before use) was added to the reaction and incubated for 2 min. Cells were washed with PBS and transferred into a 1.5 mL tube, then spun down and pelleted, and washed again once with PBS. The RNA was extracted using Direct-zol RNA Miniprep Kits (Zymo R2050) following the manufacturer's instructions (including DNA degradation) and resuspended in 88 μL water. Reverse transcription, library amplification, sequencing, and data analysis were achieved as described above for the in vitro protocol.

### NMR spectroscopy

Isotope-labeled His-MBP-tagged *S. cerevisiae* eIF4E was expressed in BL21-star *Escherichia coli* grown in M9 minimal media with ^15^N ammonium sulfate as the sole nitrogen source. Induction was performed by the addition of 1 mM IPTG when the cultures reached an OD of 0.5 and overnight incubation at 16°C. Bacteria were lysed by sonication in lysis buffer containing 500 mM NaCl, 0.5% NP-40 (Igepal), 10 mM imidazole, 20 mM HEPES pH 7.5, 10 mM 2-mercaptoethanol, clarified by centrifugation at 40,000*g*, and the clarified lysate was applied to nickel resin (QIAGEN) pre-equilibrated in wash buffer (500 mM NaCl, 20 mM imidazole, 10 mM 2-mercaptoethanol, and 20 mM HEPES pH 7.5). After three washes in wash buffer, purified protein was eluted in elution buffer containing 500 mM NaCl, 250 mM imidazole, 100 mM Na_2_SO_4_, 50 mM sodium phosphate buffer at pH 7.5, and 10 mM 2-mercaptoethanol. Protein was then dialyzed into 2 L of buffer containing 20 mM HEPES pH 7.5, 125 mM KCl, and 0.5 mM TCEP overnight at 4°C while simultaneously the affinity tags were cleaved through the addition of 1:40 (w/w) TEV protease. Dialyzed protein was applied to m^7^G cap resin (GE Healthcare) equilibrated in dialysis buffer and eluted using dialysis buffer supplemented with 0.1 mM m^7^GDP (Edery et al. 1988). Purified eIF4E protein was exchanged into 50 mM phosphate pH 6.5 and 50 mM KCl using a desalting column (Gross et al. 2003), and concentrated to 200 μM, and m^7^GTP was added to 250 μM. DDX3X 33–51 peptide (ASKGRYIPPHLRNREATKGAA) was synthesized by the HHMI Mass Spectrometry Facility at UC Berkeley and dissolved at 50 mM in water, while 4E-BP1 peptide (RIIYDRKFLMECRNSPV; amino acids 51–67) was synthesized by Elim Bio and dissolved at 50 mM in DMSO. Peptides were added to a final concentration of 1 mM. ^15^N HSQCs were acquired at 298 K using the 900 MHz spectrometer at the Central California 900 MHz NMR facility at UC Berkeley using 10% D_2_O as a lock signal. Spectra were overlaid using UCSF Sparky.

### Immunoprecipitation

For Figure 4C, HEK 293T cells transduced with DDX3X WT and mutants were washed with cold PBS (Gibco 10010049) and lysed in 500 μL of ice-cold immunoprecipitation buffer (40 mM HEPES–KOH pH 7.5, 100 mM KCl, 1 mM EDTA, 10 mM β-glycerophosphate, 10 mM NaF, 2 mM Na_3_VO_4_, 0.4% NP-40, and 1 mM PMSF). The lysates were passaged five times through a 25 gauge needle and centrifuged to remove the nuclei. RNase A (5 μg/mL, QIAGEN 19101) was added to the whole cell extracts (WCEs) and incubated on ice for 15 min. WCEs were precleared with Mouse IgG-Agarose resin (Sigma-Aldrich A0919) for 2 h at 4°C and then incubated with anti-FLAG M2 affinity gel (Millipore Sigma A2220) for 1.5 h at 4°C under continuous rotation. The beads were collected and washed five times with the same immunoprecipitation buffer, and the bead-bound proteins were resolved by SDS-PAGE and analyzed by western blotting.

For Figure 4B, HEK 293T cells transduced with DDX3X WT and mutants were washed with cold PBS, collected, and lysed in NP-40 buffer (150 mM NaCl, 1% IGEPAL, 50 mM Tris-Cl p.H. 7.5) with protease inhibitor (Roche 04693132001, cOmplete EDTA-free Protease Inhibitor Cocktail). Lysate was pipetted up and down, without vortexing. Samples were placed 45 min at 4°C and then spun down in a centrifuge at 4700 rpm for 2 min. The supernatant was transferred to new tubes and treated with MNase (NEB M0247S) at 1 μL/200 μL lysate for 30 min on the rotor at RT. RNasin (Fisher PRN2615), 4× the amount of MNase, was used to stop the reaction for 15 min at RT. Anti-FLAG M2 Magnetic Beads (Sigma M8823) were added and used to precipitate FLAG according to protocol (M8823, Millipore). Elution was achieved with 3× FLAG peptide (F4799, Sigma-Aldrich,). Briefly, 30 μL of FLAG peptide (150 μL/mL in TBS with protease inhibitor) was added to the tube and placed on the rotor for 30 min. The supernatant was collected and resolved by SDS-PAGE and analyzed by western blotting.

### Western blotting

Primary antibodies used in this study include rabbit polyclonal anti-DDX3X (custom made by Genemed Synthesis using peptide ENALGLDQQFAGLDLNSSDNQS) (Fig. 4), anti-actin HRP (Santa Cruz Biotechnology sc-47778), anti-FLAG HRP (Sigma A8592), anti-eIF4A1 antibody (ab31217), anti-eIF4G1 antibody (Cell Signaling Technologies 2498), anti-eIF4E antibody (Cell Signaling Technologies 9742), anti-ribosomal protein S11/RPS11 (ab175213), anti-RPL10A (Abcam ab174318). Band quantification in Supplemental Figure S5 was conducted with ImageJ following the protocol by Ohgane and Yoshioka (2019).

### Microscopy

For microscopy in Figure 1, HCT116 degron cells transduced with HART-ODC1 or HART-RAC1 were plated in a 384 well plate (PerkinElmer 6057302) and were treated with AUX or DMSO for 48 h. Media was removed and 50 μL of 4% PFA in PBS was added to each well for 15 min and incubated at RT. PFA solution was removed and the cells were washed with 100 μL PBS three times using a BioTek EL406. Cells were incubated with 50 μL 1× DAPI (Biotium 40043) in PBS for 5 min at RT. Cells were washed with 100 μL PBS three times using a BioTek EL406 and imaged using an InCell Analyzer 6500HS (GE), taking three fields-of-view per well. HART ratio was calculated as the ratio between the mCherry and GFP channels.

For microscopy in Supplemental Figure S4, HEK 293T cells were grown in 96 well plates. When they reached 70% confluency, media was removed and cells were washed once with PBS. Fifty microliters of 4% PFA in PBS was added to the well for 10 min at RT. Cells were washed three times with ice-cold PBS and then incubated for 10 min in PBS containing 0.1% Triton X-100 at 4°C by rocking. Cells were washed in PBS three times for 5 min at 4°C by rocking and then incubated with 1% BSA PBST solution for 30 min at 4°C by rocking. Cells were incubated in diluted antibody (ANTI-FLAG, 1:1000, Sigma-Aldrich F1804) in 1% BSA PBST overnight at 4°C by rocking. Cells were washed in PBS three times for 5 min at 4°C by rocking and then incubated by rocking with secondary antibody (Goat-anti-Mouse 488, 1:2000, Invitrogen A-11008) in 1% BSA PBST for 1 h at RT in the dark. Cells were washed in PBS three times for 5 min at 4°C by rocking in the dark. 1× DRAQ7 (Thermo Fisher Scientific D15106) in PBS was added to the cells for 15 min by rocking, followed by one wash with PBS. Images were taken with a confocal microscope at a 20×–40× magnification.

## SUPPLEMENTAL MATERIAL

Supplemental material is available for this article.

## Figures and Tables

**Kevin Wilkins:**
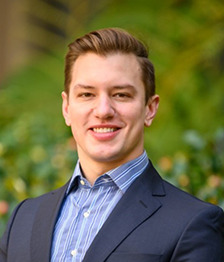


## MEET THE FIRST AUTHOR

***Meet the First Author(s)* is an editorial feature within *RNA*, in which the first author(s) of research-based papers in each issue have the opportunity to introduce themselves and their work to readers of *RNA* and the RNA research community. Kevin Wilkins is the first author of this paper, “A novel reporter for helicase activity in translation uncovers DDX3X interactions.” Kevin obtained his doctorate in Biomedical Sciences at UCSF, where he studied the role of DDX3X in mRNA translational regulation in the laboratory of Dr. Stephen Floor.**

**What are the major results described in your paper and how do they impact this branch of the field?**

In the present study, we developed a fluorescent-based and lentivirus-compatible reporter, which we term HART, to measure the activity of DDX3X in 5′ UTR regulation. We used HART to determine the impact of 5′ UTR structural changes on DDX3X sensitivity as well as to identify DDX3X residues 38–44 as contributors to the association between DDX3X and the translational machinery. We further showed that both this interaction and DDX3X's helicase activity are necessary for DDX3X's role in ribosome scanning and cell growth. HART is compatible with lentiviral systems and applicable to several in vitro and in vivo systems, its readout is measurable both by flow cytometry and microscopy, and it is adaptable to RNA helicases other than DDX3X. We expect it to be useful in various future experiments, such as drug and small-molecule screens to identify modulators of helicase activity as well as library screens to identify features that render 5′ UTR dependent on helicases for regulation.

**What led you to study RNA or this aspect of RNA science?**

I first became interested in RNA in college when grappling with the fact that the noncoding 97% of the human genome is far from “junk DNA” as previously believed, but instead quite crucial to understanding all aspects of human biology. Originally, I intended to investigate the freshest and flashiest noncoding RNA on the scene, such as lnc- or snoRNAs, but meeting Stephen and joining his laboratory opened my eyes to the immense potential and impact to be found in the less novel, yet unendingly surprising, mRNA. Ever since, RNA has not stopped fascinating me with its versatility, omnipresence in biological processes, structural and functional diversity, and therapeutical potential.

**What are some of the landmark moments that provoked your interest in science or your development as a scientist?**

Learning about the process of evolution by random mutations and natural selection in middle school was an eye-opening experience. Nature's staggering biodiversity and complexity always fascinated me as a kid, and finding out that its origin was elegantly simple was a revelation. When I later picked up *On the Origin of Species*, I expected it, a monumental undertaking and a landmark work, to be a complicated and intense read. To my delight, Darwin's prose is approachable and clear, and although not necessarily simple, it is elegant in its argumentation and exhaustive in its explanation. His unassailable logic and the straightforward presentation of the overwhelming evidence result in a read both enjoyable and persuasive as well as a template for all scientists and intellectuals on how to disseminate ideas—even radical ones—in a convincing and sound manner.

**If you were able to give one piece of advice to your younger self, what would that be?**

Scientists often encounter endless setbacks, especially in the laboratory where experiments frequently fail despite days, weeks, or months of effort. To combat this daunting challenge, I would advise both my younger self and aspiring scientists to always remember why we pursue this path. The pursuit of scientific knowledge and the advancement of understanding are immensely valuable endeavors. Even if 90% of our laboratory work ends in failure, the 10% success makes the struggle worthwhile. Furthermore, the ubiquity of failures in the laboratory serves as both solace and motivation, encouraging us to rely on each other for guidance and support.

**Are there specific individuals or groups who have influenced your philosophy or approach to science?**

As I progress in my scientific career, I find myself agreeing with Karl Popper's epistemology, that is, scientists do not possess the ability to prove absolute truths beyond doubt. Rather, scientific theories are only predictive models with varying accuracy, and they are provisionally accepted as valid only until they are falsified or refined through experimentation. His perspective liberates science from dogmatism, ensuring that no idea remains immune to questioning or revision upon further evidence. Good scientists accept this inherent uncertainty not as a limitation but as a springboard toward embracing a critical attitude toward their peers’ theories as well as their own. The invalidation of one's theory should not be regarded as a personal insult or a challenge to one's reputation, but rather as part of the vital process of the scientific method. This constant process of mutual critique and experimental falsification assures constant improvement in the predictive accuracy of scientific hypotheses, rendering science and technology a more effective tool to humanity's benefit. My doctoral adviser, Dr. Stephen Floor, has been the single biggest influence on my scientific development. In addition to his intellectual acumen and avid curiosity, I admire his approach toward the broader scientific community, which has always been guided by a pursuit of openness and honest communication in the belief that science is strengthened by transparency and the free dissemination of ideas and data.
